# Complete Plastome Sequences from *Glycine syndetika* and Six Additional Perennial Wild Relatives of Soybean

**DOI:** 10.1534/g3.114.012690

**Published:** 2014-08-25

**Authors:** Sue Sherman-Broyles, Aureliano Bombarely, Jane Grimwood, Jeremy Schmutz, Jeff Doyle

**Affiliations:** * Cornell University, Department of Plant Biology, Ithaca, New York 14853; † Hudson Alpha Institute for Biotechnology, Huntsville, Alabama 35806

**Keywords:** incongruence, divergence dates, repetitive sequences, inversions

## Abstract

Organelle sequences have a long history of utility in phylogenetic analyses. Chloroplast sequences when combined with nuclear data can help resolve relationships among flowering plant genera, and within genera incongruence can point to reticulate evolution. Plastome sequences are becoming plentiful because they are increasingly easier to obtain. Complete plastome sequences allow us to detect rare rearrangements and test the tempo of sequence evolution. Chloroplast sequences are generally considered a nuisance to be kept to a minimum in bacterial artificial chromosome libraries. Here, we sequenced two bacterial artificial chromosomes per species to generate complete plastome sequences from seven species. The plastome sequences from *Glycine syndetika* and six other perennial *Glycine* species are similar in arrangement and gene content to the previously published soybean plastome. Repetitive sequences were detected in high frequencies as in soybean, but further analysis showed that repeat sequence numbers are inflated. Previous chloroplast-based phylogenetic trees for perennial *Glycine* were incongruent with nuclear gene–based phylogenetic trees. We tested whether the hypothesis of introgression was supported by the complete plastomes. Alignment of complete plastome sequences and Bayesian analysis allowed us to date putative hybridization events supporting the hypothesis of introgression and chloroplast “capture.”

The flowering plant (angiosperm) chloroplast genome (plastome) has a wide range of uses in plant biology. Much of this is due to its behavior as a single phylogenetic unit of tightly linked genes, typically comprising 125 and 160 kb. In the majority of angiosperms, the plastome is uniparentally (generally maternally) inherited (Corriveau and Coleman 1988). Plastome sequences are useful for species identification (Nock *et al.* 2011; Kane *et al.* 2012) and biotechnology applications (Sabir *et al.* 2014; Ruhlman and Jansen 2014), and have a long history of utility for phylogenetic inference (Palmer *et al.* 1988b; Jansen *et al.* 2007; Ruhfel *et al.* 2014). Plastome sequence data combined with genes from the mitochondrion and nucleus can provide the ability to resolve ancient, higher-order relationships among genera that have remained unresolved until adequate data became available (Soltis *et al.* 2011). At the genus level and below, uniparentally inherited plastome data can indicate reticulate evolution when incongruence between plastome-derived trees do not agree with other data such as morphology or nuclear loci (Rieseberg and Soltis 1991).

Angiosperm chloroplast sequences generally evolve more slowly than angiosperm nuclear sequences (Palmer 1990). Organelle size, gene content, and gene order have remained quite conserved among land plants as compared with mitochondrial genome sequences. The typical chloroplast genome, comprising approximately 150 kb, is composed of four regions, a large single copy (LSC) region, and a small single copy region (SSC), separated by a pair of large inverted repeats (IR). Rearrangements in chloroplast gene order are generally found in taxa that have one of the following qualities: changes in the size of the IRs or complete loss of one copy of the repeat; a high frequency of small dispersed repeats; biparental chloroplast inheritance; or complete or near-complete absence of photosynthesis (Wicke *et al.* 2011). Although inversions are not common in angiosperm chloroplasts, there are several families that are known for having a number of chloroplast genome inversions: Geraniaceae (Weng *et al.* 2014; Guisinger *et al.* 2011); Onagraceae (Greiner *et al.* 2008); Campanulaceae (Haberle *et al.* 2008); Asteraceae (Kim *et al.* 2005); and Leguminosae (Cai *et al.* 2008).

The Leguminosae or legume family is the third largest land plant family. It has been divided into three subfamilies, only two of which, Mimosideae and Papilionoideae, are monophyletic (LPWG: *et al.* 2013). Research interests centered on the papilionoids are driven by the wide array of grain, oil, and forage legumes in the subfamily, encompassing at least 24 economically important genera from peanuts to beans (Cannon *et al.* 2009). Prior to the current study, there were 17 complete plastome sequences published from 14 papilionoid legume genera (Figure 1). One of the largest clades of the Papilionoideae is characterized by the loss of one copy of the IR and is thus called the “inverted repeat loss (or lacking) clade” (Wojciechowski *et al.* 2000). One genistoid chloroplast genome has recently been published, *Lupinus luteus*, European yellow lupine (Martin *et al.* 2014). Chloroplast genomes from species representing eight genera in the IRLC have been sequenced: *Trifolium aureum*; *T. grandiflorum*; *T. repens and T. subterraneum* (clover); *Medicago truncatula* (barrel medic); *Cicer arietinum* (chickpea); *Pisum sativum* (pea); *Lathyrus sativus* (grass pea); *Glycyrrhiza glabra* (licorice); and *Lens culinaris* (lentil) and *Vicia faba* (broad bean) (Sabir *et al.* 2014; Saski *et al.* 2005; Cai *et al.* 2008; Jansen *et al.* 2008; Magee *et al.* 2010). The IRLC is sister to the clade that includes the model legume *Lotus*, which retains both IRs (Kato *et al.* 2000); together, these clades comprise the Hologalegina (Figure 1). Sister to Hologalegina is the millettioid clade (Wojciechowski *et al.* 2004), from which complete chloroplast genome sequences have been obtained from *Milletia pinnata* (= *Pongamia pinnata*, pongam oiltree), as well as three phaseoloid genera, *Vigna radiata* (mung bean), *Phaseolus vulgaris* (common bean), and *Glycine max* (soybean) (Saski *et al.* 2005; Tangphatsornruang *et al.* 2010; Guo *et al.* 2007; Kazakoff *et al.* 2012).

**Figure 1:**
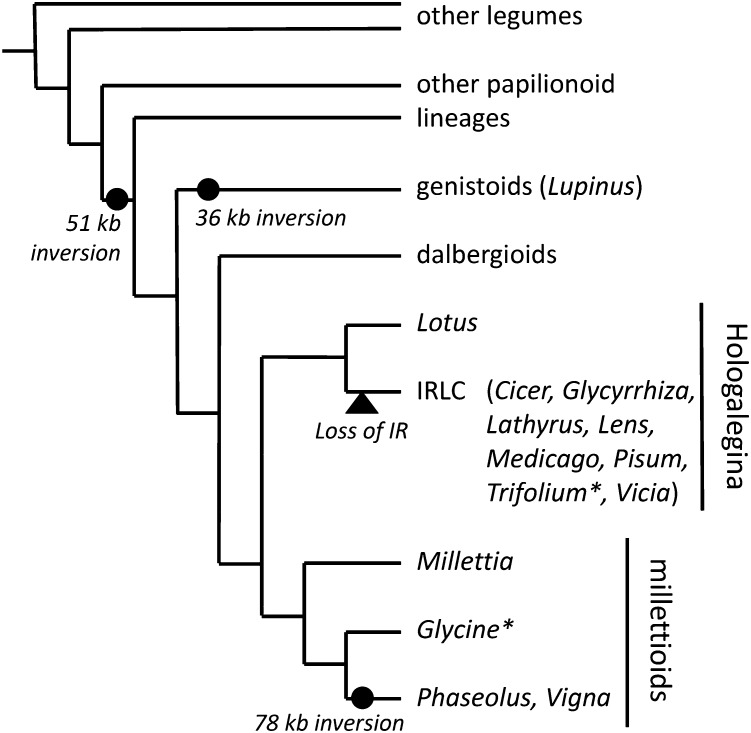
Partial representation of legume phylogeny. Fourteen genera with sequenced plastomes are named. Asterisks indicate genera with multiple species with plastome sequences.

In the legume family, a 51-kb inversion is shared by most members of subfamily Papilionoideae (Doyle *et al.* 1990b, 1996; Palmer *et al.* 1988a). This inversion is present in *Glycine* chloroplast genomes and occurs in the LSC region, changing the gene order between *trnK* and *accD*. Three additional inversions have been reported, a newly described 36-kb inversion within the 51-kb inversion is present in *Lupinus* and other genisotoids (Martin *et al.* 2014), a 78-kb inversion shared by several closely related genera including *Phaseolus* and *Vigna* (Guo *et al.* 2007; Tangphatsornruang *et al.* 2010; Bruneau *et al.* 1990), and a 5.6-kb inversion reported in *Milletia* (Kazakoff *et al.* 2012). The 78-kb inversion spans almost the entire LSC between *trnH* and *rps19*, returning the genes in the 51-kb inversion to the order found in most land plants (Guo *et al.* 2007). This change in gene order has also been attributed to the expansion and contraction of the IR, leaving the gene order as described in papilionoids with the 51-kb inversion but changing the genes bordering the IR (Tangphatsornruang *et al.* 2010; Perry *et al.* 2002).

Legume chloroplasts also show variation in the presence and absence of genes. All legumes are missing two chloroplast encoded genes, *infA* and *rpl22* (Doyle *et al.* 1995), and both have nuclear copies targeted to the chloroplast (Gantt *et al.* 1991; Millen *et al.* 2001). Loss of *rps16* from the chloroplast has been reported in a number of legume lineages excluding *Glycine*. The mitochondrial copy is dual-targeted to both the mitochondria and chloroplast (Jansen *et al.* 2008; Sabir *et al.* 2014). Intron losses in *clpP* and *rps12*, found in IRLC lineage (Jansen *et al.* 2008), are not detected in *Glycine* (Saski *et al.* 2005).

The genus *Glycine* includes at least 28 species divided into two subgenera. The annuals include *G. soja* and the cultivated soybean, *G. max*, which are native to eastern Asia, whereas the majority of species are perennials found in Australia. Early investigations grouped *Glycine* species into “genome groups” (designated by letters A–I) based on the fertility of artificially produced hybrids and the degree to which meiotic chromosomes paired (Singh and Hymowitz 1985). These data, combined with isozyme data and sequences of two nuclear loci [the nuclear ribosomal gene cistron internal transcribed spacer (nrDNA ITS) and the low copy gene histone H3D], have been used to delimit nine genome groups as reviewed by Ratnaparkhe *et al.* (2011).

Chloroplast data from annual and perennial *Glycine* species have been used in genetic diversity studies and in phylogenetic studies (Doyle *et al.* 1990b, c; Sakai *et al.* 2003; Xu *et al.* 2000, 2001, 2002) including investigations of neopolyploidy in perennial taxa (Doyle *et al.* 1990a, 2004a). For the perennial subgenus as a whole, Doyle *et al.* (1990b) identified three major clades, termed “plastome groups,” which showed varying degrees of agreement with nuclear genome groups. The B-plastomes and C-plastomes were found to characterize species of the B-genome and C-genome groups, respectively, although later work revealed incongruence between nuclear and chloroplast phylogenies within the B-genome group itself (Doyle *et al.* 1999). The A-plastome group included chloroplast genomes from all of the remaining species in the subgenus. The majority of these species belonged to the histone H3D clade comprising the A-genomes, D-genomes, E-genomes, H-genomes, and I-genomes, a result broadly congruent between chloroplast and nuclear data; however, subsequent studies suggested little agreement between genome groups and groupings within the A-plastome clade (J. T. Rauscher, A. H. D. Brown, and J. J. Doyle, unpublished data). The most obvious disagreement between nuclear and chloroplast phylogenies was the placement of *G. falcata*, the sole species of the F-genome, which in the histone H3D phylogeny was sister to all other perennials, but in the chloroplast phylogeny was strongly supported as part of the A-plastome clade. A sister relationship between *G. falcata* and other perennial *Glycine* agrees with the distinctiveness of this species (Doyle *et al.* 1996, 2004a).

In this study we describe complete plastome sequences from seven perennial *Glycine* species and compare these results to *G. max* (G-genome). We describe the *G. syndetika* plastome in detail as a representative genome of the perennial *Glycine* species. *Glycine syndetika* is currently the perennial species with the most extensive nuclear sequence available (A. Bombarely, J. Schmutz, J. Grimwood, S. A. Jackson, and J. J. Doyle, unpublished data). The perennial *Glycine* chloroplast genomes are compared with the annual soybean, *G. max* (Saski *et al.* 2005) and *Phaseolus vulgaris* (Guo *et al.* 2007), as well as other closely related legumes *Milletia pinnata* and *Vigna radiata* (Kazakoff *et al.* 2012; Tangphatsornruang *et al.* 2010).

## Materials and Methods

Five plants of a single accession each from seven perennial *Glycine* species representing five of the nine genome groups (Table 1) were grown in the greenhouse at Cornell. Plants were placed in the dark for 48 hr prior to collecting leaf tissue. Tissue was frozen and shipped to Arizona Genomics Institute (AGI), where bacterial artificial chromosome (BAC) libraries were prepared from *Hin*dIII digests of genomic DNA. BAC ends were sequenced for all BACs. Two BACs per species were selected from the BAC libraries based on BLAST matches of perennial BAC END sequence data to the *G. max* chloroplast sequence (Supporting Information, Table S1). BAC DNA was sheared to 3 kb to 4 kb using Adaptive Focused Acoustics technology (Covaris, Woburn, MA) cloned into the plasmid vector pIK96, sequenced with Sanger technology to an average depth of 9×, and then assembled (using Phrap Version 0.990319) and finished as previously described (Ferris *et al.* 2010). Additional BACs were selected as needed to close the chloroplast circles based on BLAST to the finished sequences.

**Table 1:** Summary statistics from complete *Glycine* chloroplast sequences

Plastome Group	Genome Group	*Glycine* Species	Total	LSC	SSC	IR	Duplicated *ycf1* in IR	*rps19* IR	%GC
G	G	*G. max**^a^*	152218	83174	17896	25574	478		0.34
A	A	*G. syndetika*	152794	83844	17840	25555	463	61	0.353
A	A/D	*G. dolichocarpa*	152804	83815	17807	25591	490	61	0.353
A	A	*G. canescens*	152533	83559	17844	25565	463	61	0.353
A	D	*G. tomentella* D3	152728	83773	17829	25563	463	61	0.353
A	F	*G. falcata*	153023	84027	17846	25575	463	59	0.353
B	B	*G. stenophita*	152618	83937	17817	25432	463	61	0.353
C	C	*G. cyrtoloba*	152381	89368	17801	25505	463	61	0.353

Length in base pairs of the chloroplast and the three major divisions. Extent of border genes within the IR. GC content in each chloroplast genome.

a Saski *et al.* (2005).

DOGMA (Wyman *et al.* 2004) was used with default parameters for preliminary annotation of each sequence. The complete chloroplast sequences were arranged so that each sequence started with *trnH*. The SSC regions were arranged so that the complete *ycf1* gene followed IRb, as in the *G. max* orientation depicted in Saski *et al.* (2005). Sequences were then aligned using default parameters in Mulan (Ovcharenko *et al.* 2005), with minor adjustments to the alignment made manually. *Glycine max* gene features were downloaded from NCBI (DQ317523) and aligned against the eight sequence alignment using Sequencher (Gene Codes). BioEdit (Hall 1999) was used for manual alignment and confirmation of annotations. Analysis of the total chloroplast alignment for GC content and codon bias were performed using DNAsp (Librado and Rozas 2009).

Previously published primers were used for amplification of *trnL-trnF* (Shaw *et al.* 2005) from 11 *G. falcata* accessions. The resulting sequences were aligned by MUSCLE (Edgar 2004) and edited in BioEdit (Hall 1999). This alignment was used to determine pairwise nucleotide diversity (π) between individuals. Levels of nucleotide polymorphism were calculated using DNAsp (Librado and Rozas 2009).

Direct and palindromic repeated sequences from each chloroplast sequence were identified using REPuter (Kurtz *et al.* 2001). The number of repeats identified was limited by searching for repeats greater than 30 bp in length and with a sequence identity of 90% or better (Hamming distance of 3 as used previously by Saski *et al.* 2005). To further investigate dispersed repeat sequences, we extracted the sequence of each repeat separated by more than 1000 bp. Dispersed repeat sequences were aligned using SeqMan (version 2.2.0.56; DNA-STAR, Madison WI). In many cases, repeats listed separately in the REPuter output were from the same location, had the same sequence, and the only difference was the length; for example, a 36-bp repeat could also be listed for the same position as a 30-bp repeat. Similarly, repeats were listed as pairs and, if the repeat was found in a third or fourth location, then the original location was listed again with the third location and again with the fourth location; hence, the overall number of repeats was inflated. Repeat analysis was also performed using RepeatScout and Repeat Masker (Price *et al.* 2005; Smit *et al.* 1996) using default parameters in the MAKER 2.10 (Cantarel *et al.* 2008) genome annotation software.

A Bayesian approach was used to generate a phylogenetic tree for the chloroplast genome sequences. The phylogenetic tree was generated with BEAST (Drummond *et al.* 2012) using a prior assumption that *Glycine* perennials are a monophyletic group compared with the annual *Glycine* species (*G. max*), and that *Phaseolus vulgaris* is an outgroup to the *Glycine* genus. The node date of 19.2 million years ago (mya) for *P. vulgaris* and *Glycine* in the plastome tree was used for calibration and is based on the mean age estimated from *mat*K sequence divergence in a comprehensive legume phylogeny (Lavin *et al.* 2005). The substitution model was general time reversible (GTR). The analysis was run for a MCMC length of 10,000,000 iterations, sampling every 1000 iterations. The molecular clock was an uncorrelated relaxed clock with a log normal distribution model (Drummond *et al.* 2006). FigTree was used to plot the tree (http://tree.bio.ed.ac.uk/software/figtree/).

Percent identity across the chloroplast genomes, excluding the second IR, was visualized using the VISTA tools server (Brudno *et al.* 2003). The LAGAN shuffle option was used to facilitate inclusion of chloroplast sequences from GenBank for species with inversions not present in *Glycine* (*Phaseolus vulgaris* var. Negro Jamapa (DQ886273), *Millettia pinnata* (JN673818), and *Vigna radiata* (GQ893027). Pairwise differences in single nucleotides were recorded for each species in an alignment of *Glycine species* and *Phaseolus vulgaris* plastomes. Pairwise differences were used to calculate the nucleotide substitution rate by dividing by the length of the alignment and the divergence dates calculated with histone H3D alignments (González-Orozco *et al.* 2012; Sherman-Broyles *et al.* 2014).

## Results and Discussion

### Sequencing, size, gene content, and extent of inverted repeat regions

The two *G. falcata* BAC sequences used to create a complete chloroplast sequence were polymorphic. No other species had polymorphisms between the two BAC sequences. BAC libraries for all taxa were created by collecting tissue from up to five plants in each accession. The region of overlap between the two BACs was 86,523 bp. There were 72 segregating sites in this region, resulting in pairwise nucleotide diversity or π = 0.00083. This very low level of diversity rules out contamination with chloroplast DNAs from other *Glycine* species studied here. To determine levels of chloroplast genome polymorphism in *G. falcata*, we sequenced a noncoding region of the plastome known to be variable in *Glycine* species, the intergenic spacer between *trnL* and *trnF*, in 11 additional accessions of *G. falcata* (S. Sherman-Broyles, J. A. Doyle, and J. J. Doyle, unpublished data). We found that this sample was an order of magnitude more diverse (π = 0.00225) than the estimate from the two BACs, consistent with the two BACs representing variation among individuals in accession G1718. This could be due to polymorphisms in the chloroplast genome, or to one BAC representing the chloroplast genome and the other being derived from a recent, large nuclear integrant of plastid DNA (NuPT). Similar types of variation were found in the cultivated plant *Pelargonium* × *hortorum* (Chumley *et al.* 2006). Chumley *et al.* (2006) pointed out that *Pelargonium* has biparental inheritance of chloroplasts that may contribute to the detection of variation between individuals, but also commented that they found no evidence of heteroplasmy. In *Glycine*, maternal inheritance of chloroplasts was confirmed in *G. max* (Hatfield *et al.* 1985) and in the perennials, determined by Southern blot analysis of two synthetic allopolyploid taxa (Doyle *et al.* 1990b), so it seems more likely that there are differences between individuals rather than within a single plant. Further analysis of nuclear genome sequences is required to determine if a NuPT was sequenced (Yoshida *et al.* 2013; Michalovova *et al.* 2013).

**Figure 2:**
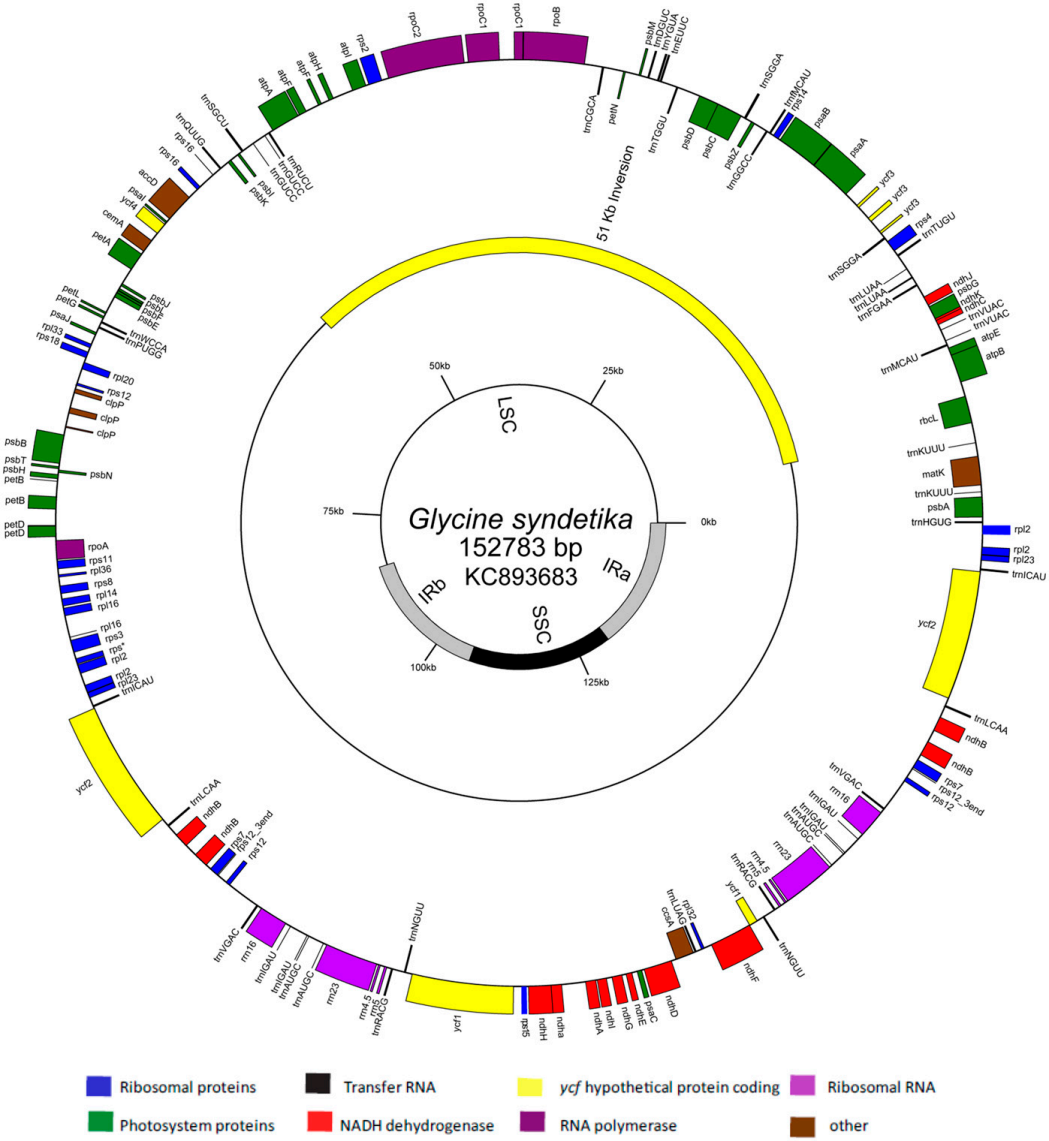
Gene map of *Glycine syndetika* chloroplast genome. Gray bars on inner circle represent the extent of the inverted repeats (IRa and IRb). Yellow bar on middle circle represents 51-kb inversion found in papillionoid legumes. Genes on the outside of the circle are transcribed in a clockwise direction, and genes on the inside of the circle are transcribed in a counter-clockwise direction.

The *G. syndetika* genome map (Figure 2) is representative of the seven perennial *Glycine* species’ chloroplast sequences discussed here. No structural rearrangements were detected among the perennial *Glycine* plastomes. The *G. syndetika* plastome sequence is 152,794 bp in length, just slightly larger than *G. max* (152,218 bp) (Saski *et al.* 2005) (Table 1). The chloroplast genome length in *Glycin*e ranges from *G. max* (with the smallest genome) to *G. falcata* at 153,023 bp (with the largest genome) (Table 1).

*Glycine* plastomes contain 111 unique genes, including 77 protein coding genes, 30 tRNA genes, and 4 rRNA genes. Six protein coding genes, seven tRNA genes, and all of the rRNA genes are completely duplicated in the IRs. The perennial *Glycine* plastomes are similar to the annual soybean plastome, with 19 genes containing introns, of which six are tRNA genes. Two genes have alternative start codons (*psbL* and *ndhD*) in all of the *Glycine* chloroplast sequences as found in other angiosperm plastomes (Sugiura 2013). The GC content in *G. max* is 34% (Saski *et al.* 2005), whereas in the perennial taxa the GC content of all species is 35% (Table 1). Codon bias is consistent with the A/T-rich aspect of angiosperm chloroplast genomes, with codons with A/T(U) in the third position being more common.

**Figure 3:**
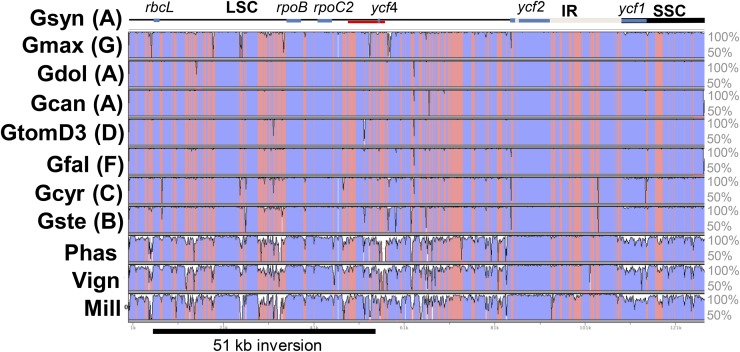
Sequence similarity plot generated by VISTA tools. Base sequence is *G. syndetika*. Intergenic regions are pink. Shuffle LAGAN option used to align *Phaseolus* and *Vigna* that have 78-kb inversions. Hypervariable region identified by Magee *et al.* (2010) is demarcated by red line. Gene with low sequence similarity to other angiosperms, *ycf4* is indicated, along with five other genes for orientation. Only one inverted repeat is shown.

The total number of genes reported above does not include *ycf4*, which in legumes is divergent from other angiosperm *ycf4* sequences (Stefanovic *et al.* 2009) and was not annotated by Dogma (Wyman *et al.* 2004) or GenBank for our submissions. Stefanovic *et al.*(2009) were the first to draw attention to the fact that the gene was present and highly divergent in legumes (Stefanovic *et al.* 2009) rather than absent, as suggested by DNA hybridization surveys (Doyle *et al.* 1995) and the *G. max* annotation (Saski *et al.* 2005). This gene is thought to be a nonessential Photosystem I assembly factor in higher plants (Krech *et al.* 2012), and this level of sequence divergence may indicate that if its function is retained in *Glycine*, it is replaced by a nuclear copy of a plastid gene (NuPT). This region flanks one of several NuPTs found in the soybean genome sequence (A. Bombarely, D. Robinson, S. Sherman-Broyles, and J. J. Doyle, unpublished data). Although *ycf4* is divergent from other legume *ycf4* sequences, there is no indication that it is a mutational hotspot in *Glycine*, as it is known to be in the legume genus *Lathyrus* (Magee *et al.* 2010).There are high levels of sequence similarity in genes between *rps16* and *cemA*, the hypervariable region in Magee *et al.* (2010) (Figure 3). Pairwise Ka/Ks values for all *Glycine* species (data not shown) are misleading because there are so few differences that ratios were based on too few polymorphic sites.

The genes that mark the beginning and end of the IR are only partially duplicated: 61 bp of *rps19* for all species except *G. falcata* (59 bp), and hypothetical chloroplast RF1 (*ycf*1), with 478 bp duplicated in *G. max*, 490 bp in *G. dolichocarpa*, and 463 bp in the remaining perennial *Glycine* taxa (Table 1). The full-length *ycf1* sequence in *G. cyrtoloba*, spanning the IRb and SSC region, has a premature stop codon in the single copy region; further analyses are necessary to determine if this is a sequencing error or an edited base, or if *ycf1* is a pseudogene in *G. cyrtoloba*. The function of *ycf1* was recently determined to be a translocon protein of the inner chloroplast membrane and in *Arabidiposis* was renamed Tic214 (Kikuchi *et al.* 2013). It has been found to have relaxed substitution rates except at its 5′ end, which may coincide with an origin of replication but might also be explained by its duplication in the IR regions, which have lower substitution rates than other chloroplast regions (Wicke *et al.* 2011).

### Repetitive sequences

Repeat sequences are of interest as a possible mechanism for inversions or as remnants of the inversion process, and thus are frequently reported in descriptions of legume plastomes and other taxa that are known to be characterized by one or more inversions.

**Figure 4:**
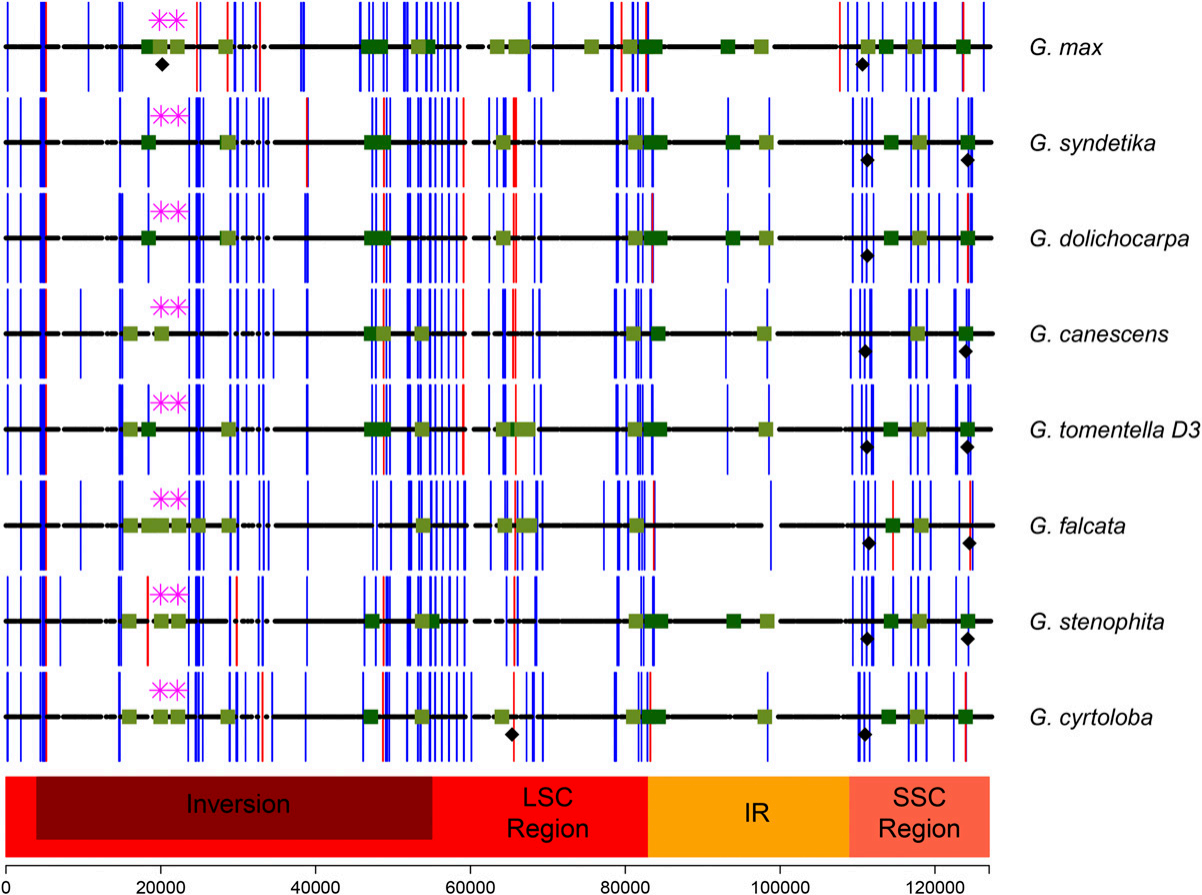
Locations of repetitive sequences in *Glycine* plastomes. Black horizontal lines represent coding regions in each plastome. Light green squares mark the location of dispersed repeat sequence 1 from REPuter. Dark green squares mark the location of 10 additional dispersed sequences. The locations of low complexity repeats are shown by blue lines. SSRs are shown by red lines as determined by RepeatScout. Asterisks represent repeats within coding sequence. Black diamonds represent repeats with sequence similarity to hAT repeat transposons.

REPuter detected 104 tandem and dispersed repeats of 30 bp in length or longer in *G. max* (Saski *et al.* 2005); similarly, in *G. syndetika* there are 103 repeats. RePuter detected the fewest (85) repeats from the *G. falcata* plastome (Table 2). The number of repeats detected in *Glycine* plastomes is much higher than the number of repeats detected previously in *Arabidopsis* (57 repeats) (Table 2) (Saski *et al.* 2005). To gain a more thorough understanding of the REPuter output, we investigated the sequence of each repeat identified. If repeat sequences were within 1000 bp of each other, then we considered them tandem repeats. If dispersed repeats occurred only in the IR regions, then we did not consider them further; if they were repeated both within and outside the IR regions, then the repeat sequences were investigated further. An alignment of all the repeats identified by REPuter and separated by more than 1000 bp showed that *Glycine* dispersed repeats with high levels of sequence similarity are found in multiple locations in the chloroplast genome. This reduced the number of unique sequences to 10 or fewer in each species and allowed us to plot their locations (Figure 4). For example, Repeat sequence 1, TATATATCTATMTATMTATAGATAGATATATAGATAT, is the most common repeat sequence and it was found in up to 11 positions in a single plastome (represented by light green squares in Figure 4). The nine remaining repeat sequences are found less frequently (four or fewer positions, depicted by dark green squares in Figure 4.) Repeats present within a coding region are depicted by an asterisk.

**Table 2:** Number and types of repeated sequences

	RePuter	Dispersed Repeats			RepeatMasker/Repeat Finder	Low Complexity	
Species	Repeat Sequences	Unique Locations	Unique Sequences	Tandem Repeats	Repeat Sequences	Low Complexity	SSR
*Arabidopsis*	57*^a^*	19	9	18			
*G. max*	104*^a^*	22	10	28	83	56	8
*G. syndetika*	103	20	5	32	95	69	7
*G. dolichocarpa*	104	20	5	29	94	69	7
*G. canescens*	86	16	5	29	95	71	5
*G. tomentella D3*	108	28	8	30	92	69	4
*G. falcata*	85	28	7	27	89	64	5
*G. stenophita*	96	19	6	31	82	58	5
*G. cyrtoloba*	101	20	6	34	78	54	6
*Phaseolus*	62	9	3	21			
*Vigna*	68	10	3	24			
*Milletia*	46	10	3	21			

Repeated sequences were detected by RePuter, RepeatMasker, and Repeat Finder. Further analysis of dispersed repeats showed that many of the sequences shared similarity, reducing the number of unique repeat sequences and locations.

a Saski *et al.* (2005).

Unlike the Geraniaceae (Guisinger *et al.* 2011) and *Oenothera* (Onagraceae) (Greiner *et al.* 2008), where inversions are often flanked by tandem or palindromic repeats, in soybean (Saski *et al.* 2005) or in the plastomes of any of the other *Glycine* species reported here, there is not a higher percentage of repeats in the area flanking the 51-kb inversion (Figure 4). The 51-kb inversion shared by most papilionoid species dates back approximately 56 mya (Lavin *et al.* 2005). The lack of repeats flanking the inversion in modern genomes sequences is not surprising considering the age of the inversions.

The sizes of repeats detected in the *Glycine* plastomes are mostly less than 50 bp, but within the range necessary for illegitimate recombination. Microhomologies as little as 16 bp in length have been shown to be sufficient for illegitimate recombination while regions of sequence similarity longer than 50 bp are necessary for homologous recombination (Maréchal and Brisson 2010). The higher incidence of repeated sequences in legume plastomes and several other taxa with inversions suggests that there may have been mutations in chloroplast DNA repair genes; this could be tested by examining candidate genes such as genes recently described as responsible for organelle stability through recombination surveillance (Maréchal and Brisson 2010).

RepeatMasker and RepeatScout (Smit *et al.* 1996; Price *et al.* 2005) detected 101 repetitive sequences in *G. syndetika* (Table 2), including short sequences with similarity to transposable elements, low-complexity repeats, and simple sequence repeats (SSRs). The locations of low complexity and SSRs are also shown in Figure 4. Comparison of *G. syndetika* with *G. dolichocarpa* indicates that the repetitive sequence profiles are identical except for a 76-bp region in *G. syndetika* with sequence similarity to hAT DNA transposon (Figure 4). All sequences with similarity to transposable elements are shorter than 506 bp and probably do not represent true transposable element insertions, but rather sequences that are coincidentally similar to transposable elements and, therefore, detected by the software.

### Evolution

**Figure 5:**
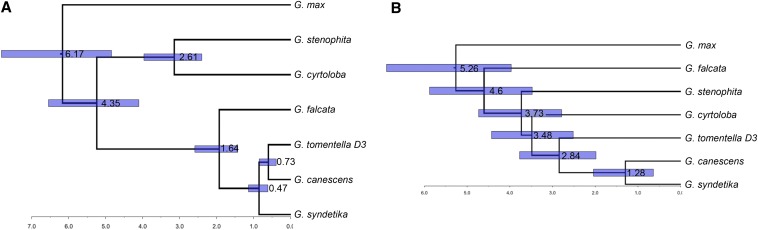
Comparison of Bayesian inference trees based on whole plastome alignment (A) and histone H3D alignment (B). Divergence dates calculated using BEAST. Bars represent the 95% HPD (highest posterior density) interval.

The alignment of the *Glycine* sequences was deposited in GenBank as Popset 514252878. Distances estimated from this alignment reveal that all species of *Glycine* outside of the B-genome, C-genome, and G-genome groups have highly similar chloroplast genome sequences, consistent with their previous recognition as the A-plastome group (Doyle *et al.* 1990b) (Table 1). A Bayesian inference tree (Figure S1), calibrated with *Phaseolus vulgaris* (Guo *et al.* 2007*)* as an outgroup, has a topology that is congruent with previous *Glycine* chloroplast gene trees (Doyle *et al.* 1990b; Sakai *et al.* 2003). All chloroplast-based trees are incongruent with topologies based on nuclear DNA sequences (Doyle *et al.* 2004b), including histone H3D (Figure 5) (Doyle *et al.* 1996b; Gonzalez-Orozco *et al.* 2012), nrDNA ITS (Rauscher *et al.* 2004), and genome-wide single nucleotide polymorphisms (D. Ilut, P. Cregan, and J. J. Doyle, unpublished data). The most conspicuous incongruence involves the placement of *G. falcata* as sister to A-genome and D-genome taxa in the chloroplast phylogeny rather than sister to all other perennial taxa as indicated by nuclear data (Figure 5).

The histone tree lacks a *P. vulgaris* sequence because, although this locus is useful for nuclear phylogenies, it is mostly intron sequence and the *P. vulgaris* sequence is too diverged to align properly. The histone tree is instead calibrated using a mean divergence date between annual and perennial *Glycine* subgenera of 5.25 mya, an average estimated from the divergence dates of several low copy nuclear genes from *G. max* and *G. tomentella* D3 (Egan and Doyle 2010; Innes *et al.* 2008). That date is similar to the 6.17 million years estimated from the BEAST plastome phylogeny and is well within the credible interval for that node (Figure 5A). These estimates of relative divergence times rather than absolute node ages are dependent on calibration using estimates from other studies and not on dates from fossils.

The plastome and nuclear gene trees differ in the depiction of the relationship between B-genome and C-genome groups. The plastome tree shows *G. stenophita* and *G. cyrtoloba* as sister to each other, sharing a common ancestor 2.61 mya, whereas the histone tree (Figure 5B) is similar to previously published trees that vary in the placement of the B and C genomes relative to each other depending on taxon sampling and the locus that was used. Regardless of the data set used, the B-genome and C-genome groups are always derived after the F-genome group and before the remaining genome groups. The presence of a B–C clade is consistent with genome-wide SNP data (D. Ilut, P. Cregan, and J. J. Doyle, unpublished data).

The strong similarities among A-plastomes lead to very recent divergence time estimates: less than 500,000 years to the common ancestor of the *G. canescens* and *G. syndetika* plastomes, whereas their histone H3D genes are estimated to have diverged 1.28 mya. Similarly, the date for the divergence of the *G. tomentella* D3 plastome from the plastomes of these A-genome species is less than 1 mya, whereas the histone divergence date is 2.84 mya. As expected, the most dramatic difference involves *G. falcata*. As sister to the remainder of the perennial subgenus in the histone H3D tree, it is estimated to have last shared a common ancestor with the other species approximately 4.6 mya, yet its chloroplast genome is estimated to have diverged from those of the other A-plastome species only 1.64 mya (Figure 5).

Doyle *et al.* (1996, 2004b) hypothesized that the plastome from the common ancestor of the entire A-plastome group was introgressed into *G. falcata* through a hybridization event and subsequent backcrossing to *G. falcata*. The plastome phylogeny divergence dates suggest that the introgression occurred as recently as 1.6 mya. The transfer of a maternally inherited chloroplast replaced the *G. falcata* plastome in what is called a chloroplast capture event (Tsitrone *et al.* 2003; Baack and Rieseberg 2007). The current range of *G. falcata* overlaps with both A-genome and D-genome species; however, little is known about the historical ranges of the taxa (E. Y. Hwang and P. Cregan, unpublished data). Based on the sample of 11 *G. falcata* accessions surveyed here for the *trnL-trnF* spacer region, there is no evidence of polymorphism involving a second, deeply coalescing chloroplast genome in this species.

In the subsequent generations, with backcrossing to *G. falcata*, the nuclear sequences in *G. falcata* have eliminated all but a few genes that demarcate the introgression event. Analysis of genome-wide SNP data from transcriptome libraries of the same taxa detected SNPs from only 80 genes out of more than 27,000 genes (< 0.3%), consistent with introgression from the A-genome into *G. falcata* (D. Ilut, P. Cregan, and J. J. Doyle, unpublished data). Even relatively recent introgression events have been reported to result in only a very low number of retained genes from the nonbackcross parent, perhaps because only genes that are selectively advantageous are retained (McKinnon *et al.* 2010). To further test the hypothesis of introgression from the common ancestor of A/D-genome ancestors into *G. falcata*, we are interested in using genotyping by sequencing, an inexpensive method for generating genome-wide markers from many individuals (Twyford and Ennos 2012).

The plastome tree has low posterior probabilities for the placement of *G. canescens*, *G. syndetika*, and *G. dolichocarpa*. The depicted tree shows the plastomes of *G. syndetika* and *G. canescens* diverging 0.43 mya, and that of *G. dolichocarpa* as slightly older at 0.50 mya. The allopolyploid, *G. dolichocarpa*, is polymorphic for chloroplast sequences from its two diploid progenitors, and this result supports previous work (J. T. Rauscher, A. H. D. Brown, and J. J. Doyle, unpublished data) in showing that the accession sampled here has the *G. syndetika* plastome. A divergence date of approximately 0.5 million years is consistent with results from nuclear genes sampled from transcriptome data (Bombarely *et al.* 2014). Hybridization events, resulting in sterile diploid offspring and fertile allopolyploids following whole genome duplication, between taxa that had diverged as many as 3 mya are common in *Glycine*, a prime example being *G. dolichocarpa*. The allopolyploid taxa lend credence to the hypothesis that a hybridization event, 1 to 3 mya, led to the capture of an A-plastome within what is probably the most earliest diverging perennial *Glycine* species, *G. falcata*.

Most differences between *Glycine* plastomes involve a handful of insertion/deletion polymorphisms, and A-plastome sequences are virtually identical to one another, with low levels of genetic diversity among taxa (Figure 3). The number of pairwise nucleotide substitutions (Table 3) can be used to estimate the plastome substitution rates in the *Glycine* taxa, if it is assumed that the annuals and perennials diverged approximately 5.25 mya (Egan and Doyle 2010; Innes *et al.* 2008) and we use the other divergence dates estimated from the histone H3D phylogeny (Figure 5). In comparisons between *G. max* and the other taxa (including not only other *Glycine* species but also *Phaseolus vulgaris*), the average substitution rate is 2.68 × 10^−9^ substitutions per site per year (Table 3). These rates are slow relative to other reported chloroplast nucleotide substitution rates (Zhong *et al.* 2009; Guo *et al.* 2007). The similarity of the substitution rates between *G. max* and the other species suggests a clock-like rate of evolution. However, when divergence dates from the nuclear gene are used, substitution rates appear much slower among the A-plastome species, averaging 7.95 × 10^−10^ substitutions per site per year. Because comparisons of these same taxa with *G. max* are clock-like, this suggests that the divergence date estimates from the nuclear genome are inappropriately high for estimating substitution rates of chloroplast genomes within this group of taxa. Recent divergence of plastomes within the A-plastome group supports a hypothesis of introgression.

**Table 3:** Pairwise SNPs and substitution rates

Rate/SNPs	*G. max*	*G. syndetika*	*G. dolichocarpa*	*G canescens*	*G. tomentella D3*	*G. falcata*	*G. stenophita*	*G. cyrtoloba*	*P. vulgaris*
*G. max*		2066	2044	2074	2104	2094	2022	2384	8234
*G. syndetika*	2.49E−09		175	210	307	638	1608	2026	8353
*G. dolichocarpa*	2.47E−09	2.22E−09		213	303	635	1596	2032	8341
*G. canescens*	2.50E−09	1.04E−09	2.70E−09		322	660	1627	2054	8331
*G. tomentella*	2.54E−09	**5.60E−10**	**5.53E−10**	**5.87E−10**		687	1640	2066	8368
*G. falcata*	2.53E−09	**8.80E−10**	**8.76E−10**	**9.11E−10**	**9.48E−10**		1640	2074	8365
*G. stenophita*	2.44E−09	2.74E−09	2.72E−09	2.77E−09	2.79E−09	2.79E−09		1284	8351
*G. cyrtoloba*	2.88E−09	3.70E−09	3.71E−09	3.75E−09	3.77E−09	3.78E−09	2.34E−09		8616
*P. vulgaris*	2.72E−09	2.76E−09	2.76E−09	2.75E−09	2.77E−09	2.77E−09	2.76E−09	2.85E−09	

The number of SNPs between species appears above the diagonal, and the substitution rate was calculated using histone H3D divergence dates. Substitution rates in bold represent A plastome taxa with substitution rates that appear slower because of inferred chloroplast capture.

## Conclusions

Our understanding of the tempo and mode of plastome evolution is enhanced as sequencing technologies have led to the increase in complete plastome sequences, including multiple species within key genera. Rearrangements observed in legumes, when only a few sequences were available, are now being shown to be specific to individual lineages at key times in legume evolution and not indicative of continuous processes leading to changes in gene order. Within *Glycine*, the chloroplast genome is very stable. Repetitive sequences do not flank the 51-kb inversion shared by the papilionoid legumes and are not as numerous as they might seem without further investigation of their location and sequence. Despite being distributed across the *Glycine* chloroplast genomes, repetitive sequences have not changed the order of genes among species. Phylogenetic analyses of complete plastome sequences corroborate previous restriction mapping studies (Doyle *et al.* 1990b) in suggesting an introgression of the A-plastome into *G. falcata*. Bayesian analysis and dating divergence allows us to hypothesize the introgression occurred 1 to 3 mya.

## Supplementary Material

Supporting Information
