# Supplementary material for: Complete Plastome Sequences from Glycine syndetika and Six Additional Perennial Wild Relatives of Soybean
Source: G3 (Bethesda). 2014 Aug 25;4(10):2023–33. doi: 10.1534/g3.114.012690 (PMC4199708; doi:10.1534/g3.114.012690)
Supplement: Supporting Information [file supp_4_10_2023__index.html]

Complete Plastome Sequences from Glycine syndetika and Six Additional Perennial Wild Relatives of Soybean — Supporting Information 

# Complete Plastome Sequences from *Glycine syndetika* and Six Additional Perennial Wild Relatives of Soybean

## Supporting Information for Sherman-Broyles *et al.*, 2014

**Files in this Data Supplement:**

- Supporting Information - Table S1 and Figure S1 (PDF, 207 KB)
- Table S1 - BAC library Information. (PDF, 109 KB)
- Figure S1 - *Glycine* plastome tree with *Phaseolus vulgaris* as the outgroup. (PDF, 97 KB)
