# Supplementary material for: Complete Plastome Sequences from Glycine syndetika and Six Additional Perennial Wild Relatives of Soybean
Source: G3 (Bethesda). 2014 Aug 25;4(10):2023–33. doi: 10.1534/g3.114.012690 (PMC4199708; doi:10.1534/g3.114.012690)
Supplement: Supporting Information [file supp_g3.114.012690_012690SI.pdf]

G3 · Genes | Genomes | Genetics

Complete plastome sequences from *Glycine syndetika*, and six additional perennial wild relatives of soybean.

Sue Sherman-Broyles*[1], Aureliano Bombarely*, Jane Grimwood§, Jeremy Schmutz§, and Jeff Doyle*

*Cornell University, Department of Plant Biology, Ithaca, NY 14853, § HudsonAlpha Institute for Biotechnology, Huntsville, AL 35806

Corresponding Author: Sue Sherman-Broyles 412 Mann Library Building, Department of Plant Biology, Cornell University, Ithaca, NY 14853 607-227-0532, sls98@cornell.edu

GenBank PopSet: 514252878 KC893632-KC893640

DOI: 10.1534/g3.114.012690

Table S1 BAC library Information. All libraries were constructed from *Hin*dIII digests of genomic DNA. Libraries available at Arizona Genomics Institute. Genbank sequence identifiers from direct submission

Glycine species	CSIRO Accession Numbers	AGI Library Name	coverage	Average insert size Kb	Chloroplast Clones Used	**GenBank Accession** Number
G. syndetika	G1300	GS_Ba	12x	168	SOG52-H04	KC893638.1
G. dolichocarpa	G1134	GD_Tba	11x	151	SOE258-B02	KC893636.1
G. canescens	G1232	GC_bC	14x	146	SOJ90-A19	KC893635.1
G. tomentella D3	G1403	GT_Dba	12x	147	SOF103-N20	KC893633.1
G. falcata	G1718	GF_BA	12x	146	SOI54-F07	KC893637.1
G. stenophita	G1974	GS_ABa	12x	149	SOK108-O10	KC893634.1
G. cyrtoloba	G1267	GC_Bb	12x	147	SOH198-B08	KC893632.1

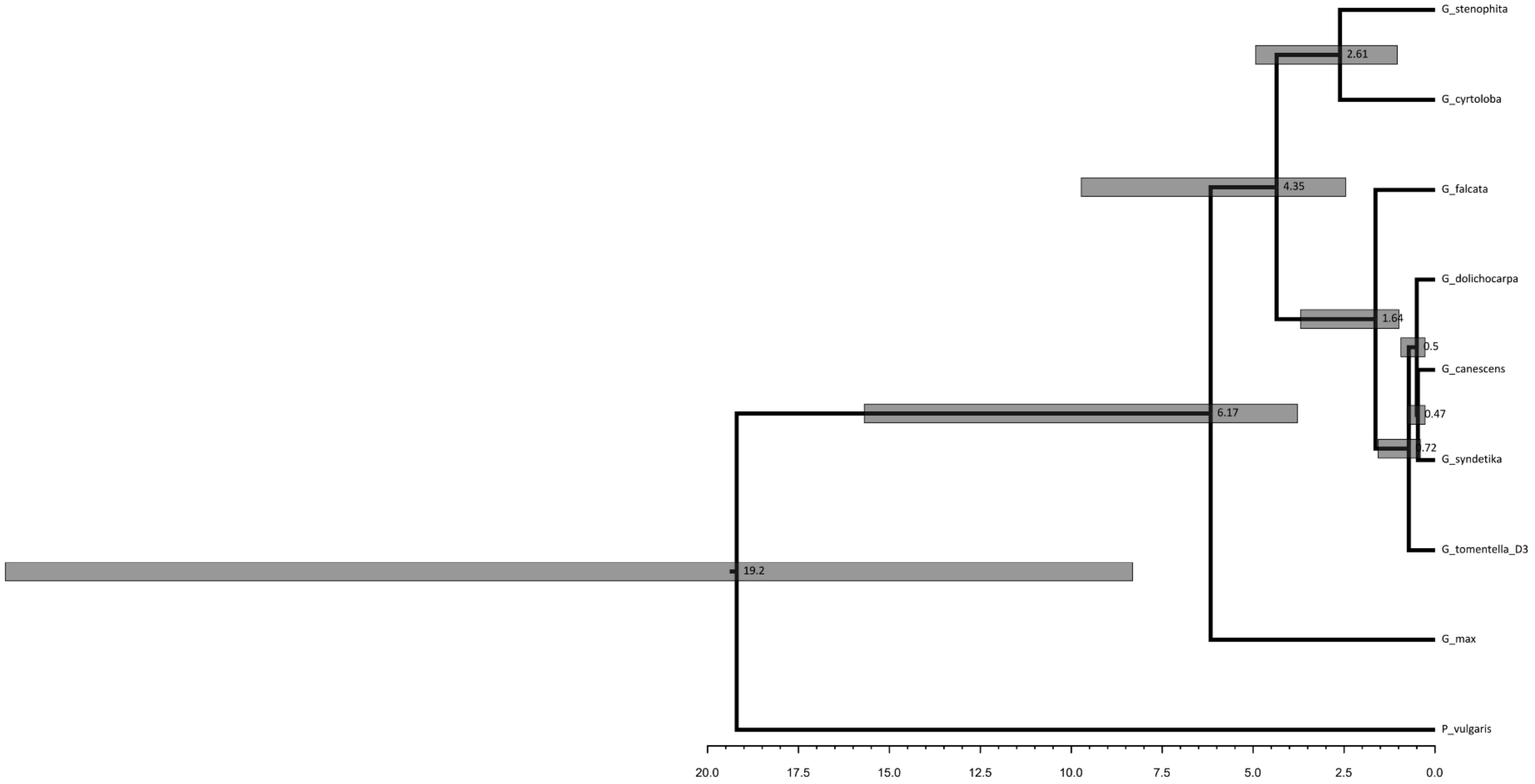

Figure S1 *Glycine* plastome tree with *Phaseolus vulgaris* as the outgroup.
