# Supplementary figures and images for: Complete Plastome Sequences from Glycine syndetika and Six Additional Perennial Wild Relatives of Soybean
Source: G3 (Bethesda). 2014 Aug 25;4(10):2023–33. doi: 10.1534/g3.114.012690 (PMC4199708; doi:10.1534/g3.114.012690)

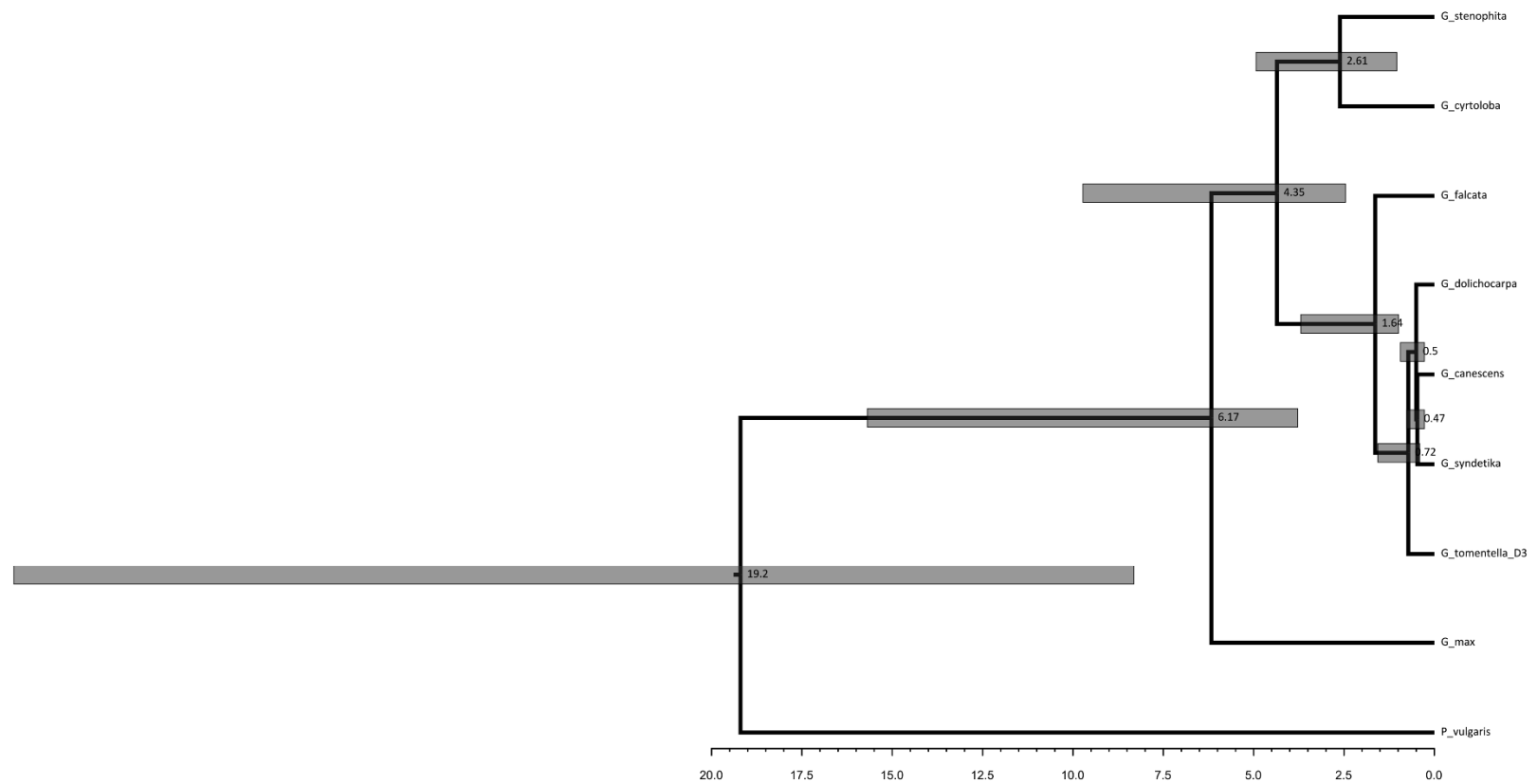

Figure S1 *Glycine* plastome tree with *Phaseolus vulgaris* as the outgroup.

Supplement: Supporting Information [file supp_g3.114.012690_FigureS1.pdf]
